# The rice orobanchol synthase catalyzes the hydroxylation of the noncanonical strigolactone methyl 4‐oxo‐carlactonoate

**DOI:** 10.1111/nph.20135

**Published:** 2024-09-19

**Authors:** Jian You Wang, Aparna Balakrishna, Claudio Martínez, Guan‐Ting Erica Chen, Salim Sioud, Angel R. de Lera, Salim Al‐Babili

**Affiliations:** ^1^ The BioActives Lab, Center for Desert Agriculture King Abdullah University of Science and Technology (KAUST) Thuwal 23955‐6900 Saudi Arabia; ^2^ Universidade de Vigo, CINBIO and Facultade de Química 36310 Vigo Spain; ^3^ The Plant Science Program, Biological and Environmental Science and Engineering Division King Abdullah University of Science and Technology (KAUST) Thuwal 23955‐6900 Saudi Arabia; ^4^ Analytical Chemistry Core Lab King Abdullah University of Science and Technology (KAUST) Thuwal 23955‐6900 Saudi Arabia

**Keywords:** hydroxyl methyl 4‐oxo‐carlactonoate, methyl 4‐oxo‐carlactonoate, MORE AXILLARY GROWTH1 (MAX1), orobanchol synthase, strigolactones

## Introduction

The plant hormone strigolactone (SL) is a main regulator of various growth and developmental processes, such as shoot branching/tillering, stem thickness, leaf senescence, and root development (Al‐Babili & Bouwmeester, 2015; Wang *et al*., 2024). Accordingly, SL‐deficient mutants, such as the rice *d17*, exhibit severe phenotypes with respect to shoot and root architecture, among others (Al‐Babili & Bouwmeester, 2015; Butt *et al*., 2018; Wang *et al*., 2024). Moreover, SLs play a pivotal role in both biotic and abiotic stress responses (Korek & Marzec, 2023). However, SLs were initially discovered in root exudates as germination signals for root parasitic weeds (Cook *et al*., 1966). Later, released SLs were identified as hyphal branching stimulants involved in the establishment of the beneficial symbiosis with arbuscular mycorrhizal fungi (AMF) under insufficient nutrient, particularly phosphate (Pi), conditions (Akiyama *et al*., 2005; Lanfranco *et al*., 2018; Wang *et al*., 2024).

**Fig. 1:**
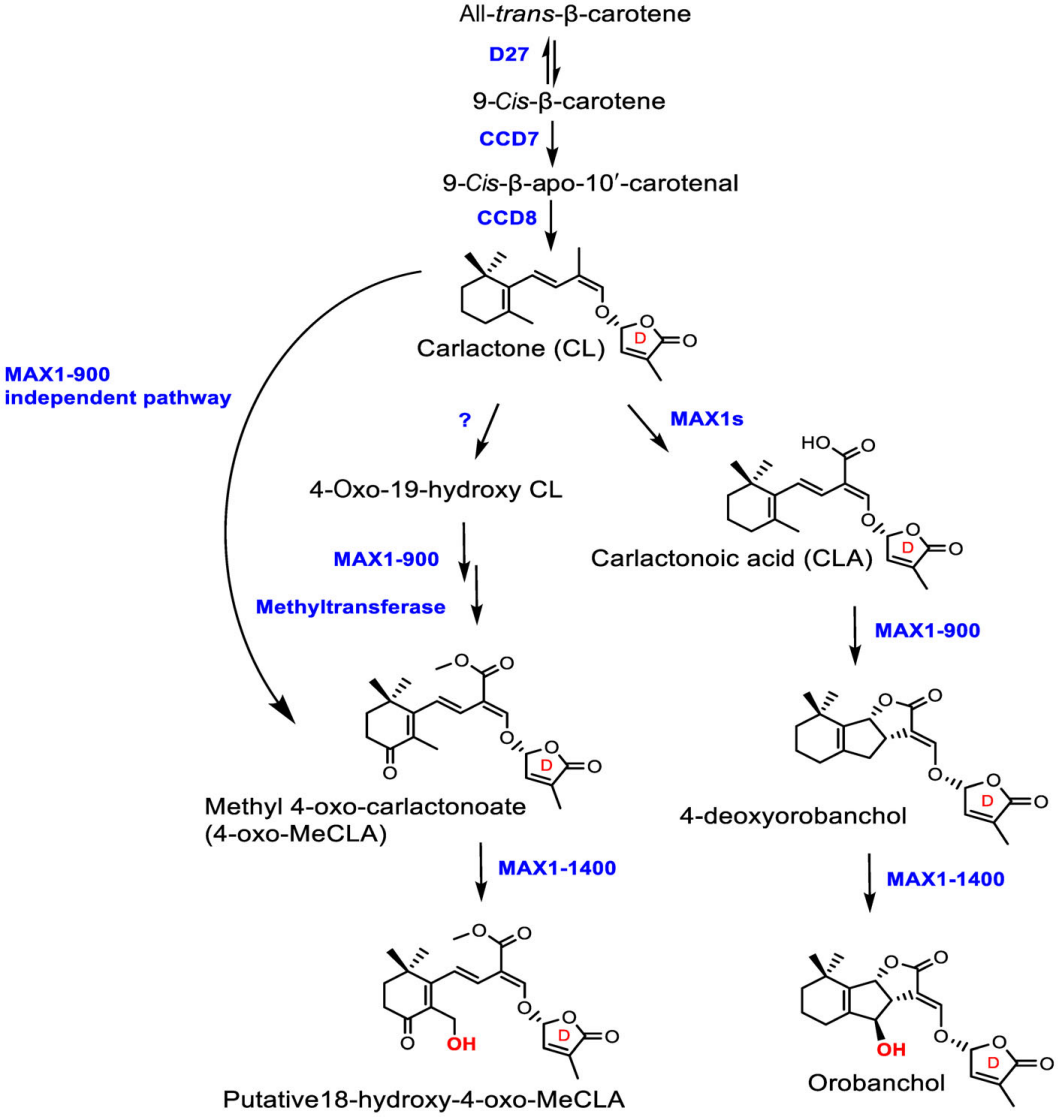
Scheme of strigolactone (SL) biosynthesis in rice. The biosynthesis of rice SLs starts with the reversible isomerization of all‐*trans*‐β‐carotene into 9‐*cis*‐β‐carotene, mediated by DWARF27 (D27). Subsequent cleavage and rearrangement reactions, mediated by CAROTENOID CLEAVAGE DIOXYGENASE 7 (CCD7) and CCD8, produce carlactone (CL). The cytochrome P450 MAX1‐900 enzyme oxygenates CL into carlactonoic acid (CLA), which is further converted into the canonical SL 4‐deoxyorobanchol (4DO). Another MAX1 homolog, MAX1‐1400, converts 4DO into orobanchol (Oro). In addition, CL is metabolized by unknown enzymes into a putative noncanonical SL, 4‐Oxo‐19‐hydroxy‐CL (CL + 30). MAX1‐900 together with an unknown methyltransferase convert 4‐oxo‐19‐hydroxy‐CL into the noncanonical SL, methyl 4‐oxo‐carlactonoate (4‐oxo‐MeCLA; previously described as methoxy‐5‐deoxystrigol isomer). The detection of 4‐oxo‐MeCLA in *Osmax1‐900* rice mutants indicates the presence of an additional, Os900‐independent and minor route for its biosynthesis. In this study, we demonstrate that OsMAX1‐1400 hydroxylates 4‐oxo‐MeCLA into the tentative 18‐OH‐4‐oxo‐MeCLA. MAX1, MORE AXILLARY GROWTH1.

Structurally, SLs are characterized by a particular feature, a butenolide ring (D‐ring; Fig. 1) linked to variable structures by an enol ether bridge in *R*‐configuration, which is essential for their biological activity (Al‐Babili & Bouwmeester, 2015; Yoneyama *et al*., 2018). Based on the presence or absence of a tricyclic lactone (the ABC‐ring; Fig. 1), they are additionally classified into canonical and noncanonical SLs, respectively (Yoneyama *et al*., 2018; Wang *et al*., 2024). The SL‐biosynthetic pathway starts with a reversible conversion of all‐*trans*‐β‐carotene into 9‐*cis*‐β‐carotene by the isomerase DWARF27 (D27). Subsequently, two carotenoid cleavage dioxygenases (CCDs), CCD7 and CCD8, convert successively 9‐*cis*‐β‐carotene into carlactone (CL), the intermediate of SL biosynthesis (Alder *et al*., 2012; Bruno *et al*., 2014, 2017; Seto *et al*., 2014; Chen *et al*., 2022). Up to now, there are more than  35 characterized natural SLs, with a structural diversity resulting from the modification of CL by cytochrome P450 monooxygenases (CYP), including the MORE AXILLARY GROWTH1 (MAX1) from the 711A clade, and other enzymes (Yoneyama *et al*., 2018; Ito *et al*., 2022; Chen *et al*., 2023; Wang *et al*., 2023, 2024). In rice, OsMAX1‐900 repeatedly oxygenates CL to produce the canonical SL 4‐deoxyorobanchol (4DO) that is further hydroxylated into orobanchol (Oro) by another CYP711A enzyme OsMAX1‐1400, the orobanchol synthase (also known as 4‐deoxyorobanchol hydroxylase) (Zhang *et al*., 2014; Ito *et al*., 2022; Chen *et al*., 2023; Fig. 1). In rice, the canonical SL pathway has been well investigated, while the biosynthesis of noncanonical SLs remains largely elusive.

Apart from its involvement in the biosynthesis of canonical SLs, OsMAX1‐900, together with unidentified methyltransferase(s), converts the putative noncanonical SL 4‐oxo‐19‐hydroxy‐CL (CL + 30) into methyl 4‐oxo‐carlactonoate (4‐oxo‐MeCLA), a tentatively identified noncanonical SL that was previously described as methoxy‐5‐deoxystrigol isomer (Yoneyama *et al*., 2018; Ito *et al*., 2022; Haider *et al*., 2023). Intriguingly, 4‐oxo‐MeCLA accumulated in the *Osmax1‐1400* and *Osmax1‐900/1400* rice mutants, but not in the *Osmax1‐900* mutant (Chen *et al*., 2023), suggesting that 4‐oxo‐MeCLA might be a substrate for MAX1‐1400, and that both MAX1‐900 and MAX1‐1400 may be involved in the biosynthesis of both canonical and noncanonical SLs. To check the supposed role of MAX1‐1400 in 4‐oxo‐MeCLA metabolism, we expressed the enzyme in yeast, incubated the isolated microsomes with different substrates, and analyzed the product formation by liquid chromatography–tandem mass spectrometry (LC‐MS/MS). We also analyzed the root exudates of *Osmax1‐900* and *Osmax1‐900/1400* rice mutants, to determine the role of MAX1‐1400 in the biosynthesis of noncanonical SLs.

## Materials and Methods

### Plant material and growth conditions

*Oryza sativa* Nipponbare *max1‐900, max1‐900/1400* (Ito *et al*., 2022; Chen *et al*., 2023), and wild‐type (WT) rice plants were grown under controlled conditions (a 12 h photoperiod, 200‐μmol photons m^−2^ s^−1^ and day : night temperature of 27°C : 25°C). All rice seeds were first surface‐sterilized in a 50% sodium hypochlorite solution for 10 min, then rinsed with sterile water, before being germinated in the dark overnight. The pregerminated seeds were placed on Petri dishes containing ½‐strength liquid Murashige & Skoog (MS) medium and incubated in a growth chamber for 7 d. After germination, the seedlings were transferred into 50 ml black falcon tubes filled with low‐Pi half‐strength modified Hoagland nutrient solution with pH adjusted to 5.8. The nutrient solution consisted of 5.6 mM NH_4_NO_3_, 0.8 mM MgSO_4_·7H_2_O, 0.8 mM K_2_SO_4_, 0.18 mM FeSO_4_·7H_2_O, 0.18 mM Na_2_EDTA·2H_2_O, 1.6 mM CaCl_2_·2H_2_O, 0.8 mM KNO_3_, 0.023 mM H_3_BO_3_, 0.0045 mM MnCl_2_·4H_2_O, 0.0003 mM CuSO_4_·5H_2_O, 0.0015 mM ZnCl_2_, 0.0001 mM Na_2_MoO_4_·2H_2_O and 0.004 mM K_2_HPO_4_·2H_2_O.

### Gene cloning

OsCYP711A3 (OsMAX1‐1400) gene is amplified from the vector pAG414GPD‐OsCYP711A3 (pYL761) (https://www.addgene.org/178300/) by using the primers, OsCYP711A3_OS1400F_Bam HI: ATTGGATCCATGGAAATCATCTCTACCGTTTTG OsCYP711A3_OS1400R2_6XHIS_ECoRI:ATTCCGCGGTTAATGATGATGATGATGatgGGCAGTATGTCTCTTGATGAC, and inserted into the vector pJKW1148 (Plasmid #119029, https://www.addgene.org/119029).

### Preparation of cytochrome P450 microsomes

*Saccharomyces cerevisiae* WAT11 yeast cells expressing the ATR1 Arabidopsis NADPH‐P450 reductase (Truan *et al*., 1993; Pompon *et al*., 1995) were cultivated in complete YPGA medium (10 g l^−1^ yeast extract, 10 g l^−1^ bactopeptone, 20 g l^−1^ glucose, and 200 mg l^−1^ adenine). Cells transformed with pYeDP60_OsCYP711A3 plasmids using a modified lithium acetate protocol (Gietz *et al*., 1992) and selected on SGI plates containing Bactocasamino acids 1 g l^−1^, yeast nitrogen base 7 g l^−1^, glucose 20 g l^−1^, and Tryptophane 20 mg l^−1^. Transformed colonies were grown in 30 ml of SGI medium at 28°C until an OD_600_ of 0.2. Cultures were then diluted at a ratio of 1 : 100 in YPGE medium (10 g l^−1^ yeast extract, 10 g l^−1^ bactopeptone, 20 g l^−1^ glucose, and 3% (v/v) ethanol) and incubate at 28°C until an OD_600_ of 0.5. Expression of OsCYP711A3 was induced by adding 10% (v/v) of a 20% (w/v) galactose solution, and cultures were then incubate at 25°C for 16 h.

A 1‐l culture of *S. cerevisiae* WAT11 cells was harvested and washed with 90 ml cold buffer TEK consisting of 50 mM Tris/HCl pH 7.5, 100 mM KCl and 1 mM EDTA. Pellets were resuspended in 10 ml cold TES buffer (50 mM Tris/HCl pH 7.5, 1 mM EDTA, 0.6 M sorbitol) containing 1% (w/v) BSA and 0.03% (v/v) β‐mercaptoethanol. Glass beads (0.45–0.50 mm diameter) were added until skimming the top of the cell suspension, and cell walls were mechanically disrupted by handshaking for 5 min in the cold room. The extracts were recovered, and the beads were washed using 1 ml of TES buffer. The extracts were centrifuged for 10 min at 3500 ***g*** and 4°C. Supernatants were then filtered using Miracloth filter (pore size 22–25 μm; Merck), and centrifuged at 10^5^ ***g*** and 4°C for 2 h. Pellets representing the microsomal preparation were resuspended in 3.5 ml cold TEG buffer TEG (50 mM Tris/HCl pH 7.5 containing 1 mM EDTA and 30% (v/v) glycerol) and frozen at −80°C.

### *In vitro* enzymatic assays with cytochrome P450 microsomes

*In vitro* assays were performed in a total volume of 500 μl of 10 mM sodium phosphate buffer (pH 7.4), 500 μM NADPH, 2 μM substrate (final concentration), 0.1% (v/v). Two‐hundred microliter microsomes were then added and the assay volume was adjusted with water. Incubations were performed for 2 h at 28°C. Assays were extracted by partitioning against two volumes of ethyl acetate. Lipophilic epiphase was dried using a vacuum centrifuge and re‐dissolved in 100% acetone for LC‐MS analysis. (*R*)‐4DO was purchased from Olchemim, Olomouc, Czech Republic.

### Chemical synthesis of methyl 4‐oxo‐carlactonoate (4‐oxo‐MeCLA)

Methyl 4‐oxo‐carlactonoate was prepared from previously described alkenylstannane (Woo & McErlean, 2019) and alkenyl iodide (Domínguez *et al*., 2012), by the Stille‐Migita‐Kosugi cross‐coupling reaction (Stille, 1986; Farina & Roth, 1996; Heravi *et al*., 2014), which took place at ambient temperature upon addition of catalytic amounts of Pd_2_dba_3_ and AsPh_3_ with CuI as additive in 59% yield. The chemical synthesis procedures and NMR characterization of 4‐oxo‐MeCLA are listed in Supporting Information Fig. S1.

### SL identification and quantification in root exudates and yeast microsomes

Collection, extraction, and analysis of SLs in rice root exudates were followed the published protocol (Wang *et al*., 2022b). Briefly, root exudates were extracted with a C_18_‐Fast Reversed‐SPE column (500 mg per 3 ml), preconditioned with 3 ml of methanol and followed with 3 ml of Milli‐Q water. After washing with 3 ml of water, SLs were eluted with 5 ml of acetone. Thereafter, the SLs‐containing fraction was concentrated to SL aqueous solution (*c*. 500 μl), followed by 1 ml of ethyl acetate extraction. Seven‐hundred‐and‐fifty microliters of SL enriched fraction was dried under vacuum. The final extract was re‐dissolved in 100 μl of 100% acetone and filtered through a 0.22 μm filter for LC‐MS/MS analysis.

The identification of SLs was performed using Vanquish UHPLC‐Orbitrap‐ID‐X Tribrid Mass Spectrometer (Thermo Scientific™, Waltham, MA, USA) with a heated‐electrospray ionization source. The IDX Orbitrap‐MS was tuned and calibrated using Pierce™ Flex Mix™ Calibration Solution according to the manufacturer's guidelines. Chromatographic separation was achieved on the Hypersil GOLD C_18_ Selectivity HPLC Columns (150 × 4.6 mm; 3 μm; Thermo Scientific™) with mobile phases consisting of water (A) and acetonitrile (B), both containing 0.1% formic acid, and the following linear gradient (flow rate, 0.5 ml min^−1^): 0–15 min, 25–100% B, followed by washing with 100% B and equilibration with 25% B for 3 min. The injection volume was 10 μl, and the column temperature was maintained at 30°C for each run. The MS conditions of H‐ESI mode were as follows: positive mode, ion source of H‐ESI, spray voltage of 3500 V, sheath gas flow rate of 60 arbitrary units, auxiliary gas flow rate of 15 arbitrary units, sweep gas flow rate of 2 arbitrary units, ion transfer tube temperature of 350°C, vaporizer temperature of 400°C, S‐lens RF level of 60, resolution of 120 000 for MS; stepped HCD collision energies of 10–50% and resolution of 30 000 for MS/MS. The MS conditions of APCI mode were as follows: ion source of APCI, Pos Ion discharge current 4 μA, sheath gas flow rate of 45 arbitrary units, auxiliary gas flow rate of 10 arbitrary units, sweep gas flow rate of 2 arbitrary units, ion transfer tube temperature of 275°C, vaporizer temperature of 400°C, RF level of 50, resolution of 120 000 for MS; stepped HCD collision energies of 10–50% and resolution of 30 000 for MS/MS. The mass accuracy of the identified compounds (accurate mass ± 5 ppm mass tolerance) was acquired using XCALIBUR software v.4.1.

Strigolactones were quantified by LC‐MS/MS using a UHPLC‐Triple‐Stage Quadrupole Mass Spectrometer (Thermo Scientific™ Altis™). Chromatographic separation was the same as above for SL identification. The MS parameters were positive ion mode, ion source of H‐ESI, ion spray voltage of 5000 V, sheath gas of 40 arbitrary units, aux gas of 15 arbitrary units, sweep gas of 2 arbitrary units, ion transfer tube gas temperature of 350°C, vaporizer temperature of 350°C, collision energy of 17 eV, CID gas of 2 mTorr. The characteristic multiple reaction monitoring (MRM) transitions (precursor ion → product ion) were 361.16 → 247.12, 361.16 → 177.05, 361.16 → 208.07, 361.16 → 97.028 for 4‐oxo‐MeCLA; 377.3 → 97.028, 377.3 → 251.13, 377.3 → 251.13, 377.3 → 331.15 for OH‐4‐oxo‐MeCLA; 360.13 → 213.07, 360.13 → 185.07, 360.13 → 97.028 for (OH‐4‐oxo‐MeCLA (−H_2_O)); 331.15 → 216.0, 331.15 → 234.1, 331.15 → 97.028 for 4‐deoxyorobanchol; 347.14 → 329.14, 347.14 → 233.12, 347.14 → 205.12, 347.14 → 97.028 for orobanchol; 333.17 → 219.2, 333.17 → 173.2, 333.17 → 201.2, 333.17 → 97.028 for putative 4‐oxo‐hydroxyl‐CL (CL + 30); 317.17 → 164.08, 317.17 → 97.028 for putative Oxo‐CL (CL + 14); 299.09 → 185.06, 299.09 → 157.06, 299.09 → 97.028 for GR24.

## Results and Discussion

We first incubated the yeast microsomes containing OsMAX1‐1400 with root exudates of WT rice grown hydroponically under constant low‐Pi conditions. As expected, we observed a significant decrease in the level of 4DO, accompanied by an enhancement in Oro content (Figs S2, S3). Intriguingly, the content of the putative 4‐oxo‐MeCLA was also remarkably reduced after incubation, indicating that 4‐oxo‐MeCLA can also be a substrate for MAX1‐1400 enzyme (Figs S2, S4).

**Fig. 2:**
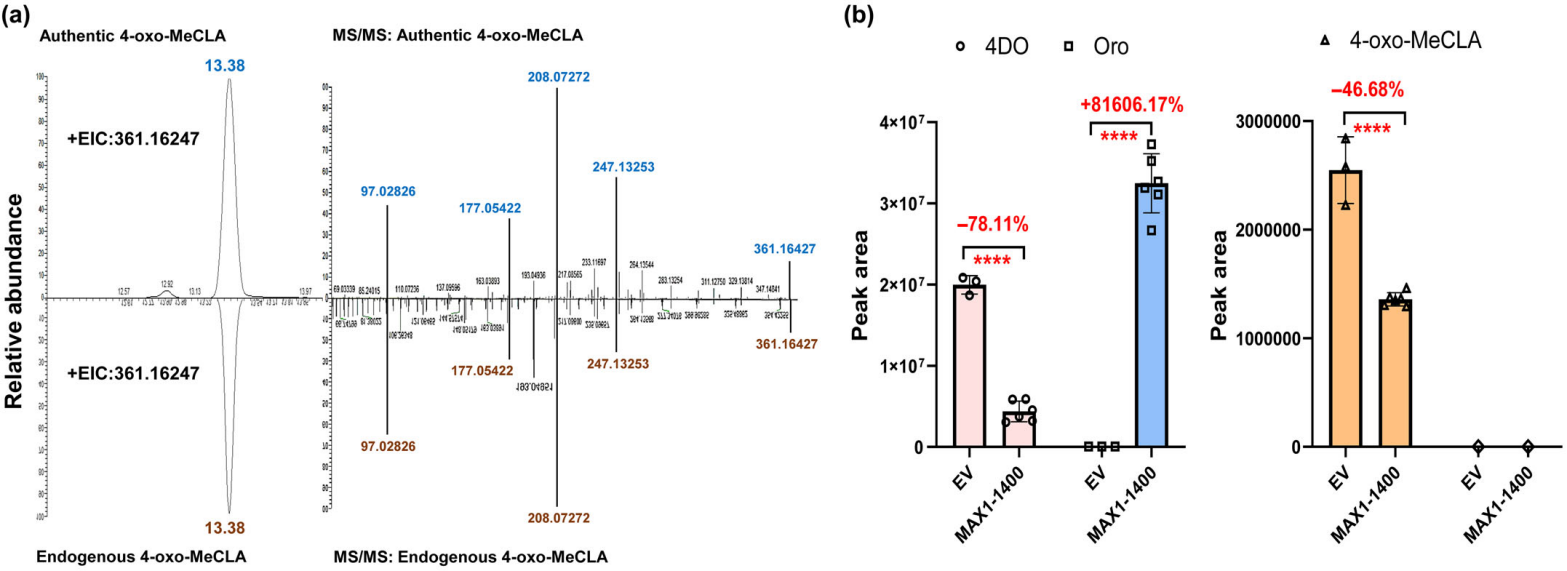
OsMAX1‐1400 converts the canonical strigolactone (SL) 4DO as well as the noncanonical 4‐oxo‐MeCLA. (a) Extracted ion chromatogram (EIC) of the identification of 4‐oxo‐MeCLA (retention time: 13.38). Identification of endogenous 4‐oxo‐MeCLA in the root exudate of wild‐type (WT) rice, based on retention time (left) and MS/MS fragmentation (right), in comparison to authentic standard. Product ion spectra derived from the precursor ion, 4‐oxo‐MeCLA, (*m/z*: 361.16247 [M + H]^+^). (b) Quantification of the conversion of 4DO and 4‐oxo‐MeCLA by yeast microsomes containing MAX1‐1400 (MAX1‐1400) and the corresponding empty vector control (EV). The decrease in 4DO and 4‐oxo‐MeCLA (left), and the increase in Oro (right) are shown in percentages in comparison to the EV. The data are presented as means ± SD of ≥ 3 biological replicates. Asterisks indicate statistically significant differences as compared to control by the two tailed unpaired Student *t* test (****, *P* < 0.0001).

For unambiguous identification of the conversion product(s), a structurally confirmed 4‐oxo‐MeCLA is required; therefore, we chemically synthesized this noncanonical SL (Fig. S5) and compared it with the endogenous compound using LC‐MS, which allowed us to unambiguously confirm it as a noncanonical rice SL (Fig. 2a). Next, we incubated the MAX1‐1400 enzyme with enantiopure (*R*)‐4‐oxo‐MeCLA (at 2 μM concentration) and used (*R*)‐4DO as a positive control. We again detected an *c.* 80% reduction in 4DO content and a significant level of Oro produced by MAX1‐1400, confirming that MAX1‐1400 is a 4DO hydroxylase (Fig. 2b; Zhang *et al*., 2014; Chen *et al*., 2023). With respect to 4‐oxo‐MeCLA, we observed *c*. 50% decrease in its content, pointing to this noncanonical SL as a further substrate of MAX1‐1400. However, we did not detect any potential product(s) in this experiment (Fig. 2b).

**Fig. 3:**
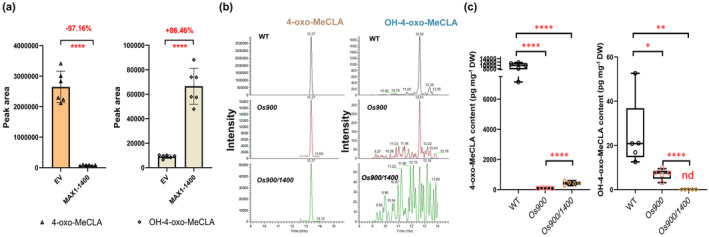
Identification and quantification of OH‐4‐oxo‐MeCLA. (a) Quantification of OH‐4‐oxo‐MeCLA upon feeding of yeast microsomes containing MAX1‐1400 with 4‐oxo‐MeCLA. The decrease in 4‐oxo‐MeCLA (left), and the increase in OH‐4‐oxo‐MeCLA (right) are shown in percentages in comparison to the empty vector control (EV). (b) Multiple reaction monitoring chromatograms showing the detection of 4‐oxo‐MeCLA and OH‐4‐oxo‐MeCLA in the pooled root exudates. OH‐4‐oxo‐MeCLA is absent in the root exudate of *Osmax1‐900/1400* mutant plants. The pool of root exudates combined 12 independent biological replicates. (c) Analysis of strigolactones (SLs) in root exudates of wild‐type (WT), *Osmax1‐900*, and *Osmax1‐900/1400* mutants grown under low‐Pi conditions. Data are presented as means ± SD of ≥ 5 biological replicates. Asterisks indicate statistically significant differences as compared to control by the two tailed unpaired Student *t* test (*, *P* < 0.05; **, *P* < 0.01; ****, *P* < 0.0001). nd, not detected.

To identify the product(s), we combined six independent microsome assays, aiming to increase the product concentration, and performed untargeted LC‐MS analysis, using void microsomes as a negative control. On the basis of the MS‐fragment at 97.02820 (mass/charge ratio (*m/z*) ± 5 ppm), which is characteristic for the D‐ring, we identified a product with a molecular formula C_20_H_25_O_7_ (*m/z*) 377.16074 as positive ion [M + H]^+^ in the atmospheric pressure chemical ionization mode (Fig. S6). By using electrospray ionization (ESI) mode, we obtained an accumulated metabolite in the MAX1‐1400 sample with a molecular formula C_20_H_24_O_6_ (*m/z*) 360.13440 ± 5 ppm as positive ion [M + H]^+^ (Fig. S7), indicating a loss of water [M + H (−H_2_O)]^+^. The shift in the molecular mass from 4‐oxo‐MeCLA (molecular formula C_20_H_25_O_6_; (*m/z*) 361.16456 ± 5 ppm as positive ion [M + H]^+^) to the product indicated that MAX1‐1400 catalyzes a hydroxylation (–OH) that leads to OH‐4‐oxo‐MeCLA (Fig. S6). To further validate this assumption, we quantified 4‐oxo‐MeCLA and OH‐4‐oxo‐MeCLA in another independent assay. Interestingly, we observed a significant increase (*c*. 85%) in OH‐4‐oxo‐MeCLA content accompanied by a *c*. 97% decrease of the substrate, demonstrating that OH‐4‐oxo‐MeCLA is a metabolite produced by MAX1‐1400 *in vitro* (Fig. 3a). Furthermore, MS/MS fragmentation allowed us to tentatively identify the product as 18‐OH‐4‐oxo‐MeCLA (Fig. S8); however, nuclear magnetic resonance (NMR) spectroscopy will be needed to precisely confirm the structure of this noncanonical SL.

Recently, we showed that 4‐oxo‐MeCLA accumulated in rice mutants, such as *Osmax1‐1400* and *Osmax1‐900/1400*, which are disrupted in *MAX1‐1400*, while it occurred in trace amounts in *Osmax1‐900* mutants (Ito *et al*., 2022; Wang *et al*., 2022a; Chen *et al*., 2023). This pattern suggests the involvement of MAX1‐1400 in metabolizing 4‐oxo‐MeCLA, and is consistent with the here described *in vitro* results. To further confirm this conclusion, we analyzed the SLs present in the WT, *Osmax1‐900*, and *Osmax1‐900/1400* mutants, excluding the potential activity of MAX1‐900 in noncanonical SL biosynthesis. As expected, none of the mutants contained detectable amounts of the canonical SLs 4DO and Oro, while both of them accumulated higher levels of the putative CL + 30 and CL + 14 (Oxo‐CL), in comparison to the WT (Fig. S9). Interestingly, we did not detect the tentative 18‐OH‐4‐oxo‐MeCLA in the root exudates of *Osmax1‐900/1400*, but this noncanonical SL was present in the exudates of *Osmax1‐900* and WT plants (Fig. 3b). In addition, we observed a higher content of 4‐oxo‐MeCLA in *Osmax1‐900/1400*, compared to *Osmax1‐900* (Fig. 3c). These results suggest that OsMAX1‐1400 catalyzes the hydroxylation of 4‐oxo‐MeCLA *in planta* and that the formation of the tentative 18‐OH‐4‐oxo‐MeCLA requires a functional MAX1‐1400 (Fig. 3c).

## Conclusion

Taken together, our work demonstrates that the rice orobanchol synthase, OsMAX1‐1400, hydroxylates 4‐oxo‐MeCLA into a tentative 18‐OH‐4‐oxo‐MeCLA *in vitro*, besides the known hydroxylation of 4DO into Oro. By analyzing the corresponding mutants, we also show that this conversion takes place *in planta*, depending on OsMAX1‐1400. Additionally, our results unarguably reveal the involvement of OsMAX1400 in noncanonical SL biosynthesis and expand the current knowledge of the enzymatic activities of MAX1s and their relation to both SL classes (Fig. 1a). The contribution of canonical SL‐biosynthetic enzymes to the formation of noncanonical SLs may occur in other plant species and be related to the evolution of these enzymes. Future work, including the elucidation of SL biosynthesis at the cellular level, will identify the function of 4‐oxo‐MeCLA and the tentative 18‐OH‐4‐oxo‐MeCLA in rice and reveal the biological significance of this conversion. This may first require the elucidation of the biosynthesis of CL + 30, the precursor of 4‐oxo‐MeCLA. In any case, the accumulation of 4‐oxo‐MeCLA and/or the lack of tentative 18‐OH‐4‐oxo‐MeCLA might be a reason for the reduced AM symbiosis observed in *Osmax1‐1400* mutants (Chen *et al*., 2023).

## Competing interests

None declared.

## Author contributions

SA‐B and JYW proposed the concept and designed the experiments. JYW, AB and G‐TEC discussed and conducted experiments. CM and ARL synthesized 4‐oxo‐MeCLA. SS and JYW analyzed MS/MS data and proposed the structure. JYW and SA‐B analyzed and discussed the data. JYW and SA‐B wrote the manuscript. All authors read, edited and approved the manuscript.

## Supporting information


**Fig. S1** Experimental procedures for the total synthesis of methyl 4‐oxo‐carlactonoate.
**Fig. S2** MAX1‐1400 potentially metabolizes both canonical and noncanonical SLs.
**Fig. S3** Detection of 4DO and Oro in root exudates feeding experiments with recombinant MAX1‐1400 in yeast microsomes.
**Fig. S4** Detection of 4‐oxo‐MeCLA in root exudates feeding experiments with recombinant MAX1‐1400 in yeast microsomes.
**Fig. S5** Synthesis of methyl 4‐oxo‐carlactonoate (4‐oxo‐MeCLA).
**Fig. S6** Identification of OH‐4‐oxo‐MeCLA.
**Fig. S7** Detection of OH‐4‐oxo‐MeCLA in 4‐oxo‐MeCLA feeding experiments with recombinant MAX1‐1400.
**Fig. S8** Proposed structure of tentative 18‐OH‐4‐oxo‐MeCLA based on mass fragmentation.
**Fig. S9** SL quantification of *Os900*‐KO line and *Os900/1400*‐KO mutants.
Please note: Wiley is not responsible for the content or functionality of any Supporting Information supplied by the authors. Any queries (other than missing material) should be directed to the *New Phytologist* Central Office.

## Data Availability

All data needed to evaluate the conclusions in the paper are present in the paper (Figs 1, 2, 3) and/or the Supporting Information (Figs S1–S9).
