# Supplementary material for: The rice orobanchol synthase catalyzes the hydroxylation of the noncanonical strigolactone methyl 4‐oxo‐carlactonoate
Source: New Phytol. 2024 Sep 19;244(6):2121–6. doi: 10.1111/nph.20135 (PMC11579440; doi:10.1111/nph.20135)
Supplement: Supplementary file 1 — Fig. S1 Experimental procedures for the total synthesis of methyl 4‐oxo‐carlactonoate. Fig. S2 MAX1‐1400 potentially metabolizes both canonical and noncanonical SLs. Fig. S3 Detection of 4DO and Oro in root exudates feeding experiments with recombinant MAX1‐1400 in yeast microsomes. Fig. S4 Detection of 4‐oxo‐MeCLA in root exudates feeding experiments with recombinant MAX1‐1400 in yeast microsomes. Fig. S5 Synthesis of methyl 4‐oxo‐carlactonoate (4‐oxo‐MeCLA). Fig. S6 Identification of OH‐4‐oxo‐MeCLA. Fig. S7 Detection of OH‐4‐oxo‐MeCLA in 4‐oxo‐MeCLA feeding experiments with recombinant MAX1‐1400. Fig. S8 Proposed structure of tentative 18‐OH‐4‐oxo‐MeCLA based on mass fragmentation. Fig. S9 SL quantification of Os900‐KO line and Os900/1400‐KO mutants. Please note: Wiley is not responsible for the content or functionality of any Supporting Information supplied by the authors. Any queries (other than missing material) should be directed to the New Phytologist Central Office. [file NPH-244-2121-s001.pdf]

New Phytologist

***New Phytologist* Supporting Information**

Article title: The rice orobanchol synthase catalyzes the hydroxylation of the non-canonical strigolactone methyl 4-oxo-carlactonoate

Authors: Jian You Wang, Aparna Balakrishna, Claudio Martínez, Guan-Ting Erica Chen, Salim Sioud, Angel R de Lera, and Salim Al-Babili

Article acceptance date: 29 August 2024

The following Supporting Information (SI) is available for this article:

Fig. S1 Experimental procedures for the total synthesis of methyl 4-oxo-carlactonoate.

Fig. S2 MAX1-1400 potentially metabolizes both canonical and non-canonical SLs.

Fig. S3 Detection of 4DO and Oro in root exudates feeding experiments with recombinant

MAX1-1400 in yeast microsomes.

Fig. S4 Detection of 4-oxo-MeCLA in root exudates feeding experiments with recombinant

MAX1-1400 in yeast microsomes.

Fig. S5 Synthesis of methyl 4-oxo-carlactonoate (4-oxo-MeCLA).

Fig. S6 Identification of OH-4-oxo-MeCLA.

Fig. S7 Detection of OH-4-oxo-MeCLA in 4-oxo-MeCLA feeding experiments with recombinant

MAX1-1400.

Fig. S8 Proposed structure of tentative 18-OH-4-oxo-MeCLA based on mass fragmentation.

Fig. S9 SL quantification of Os900-KO line and Os900/1400-KO mutants.

SI REFERENCES

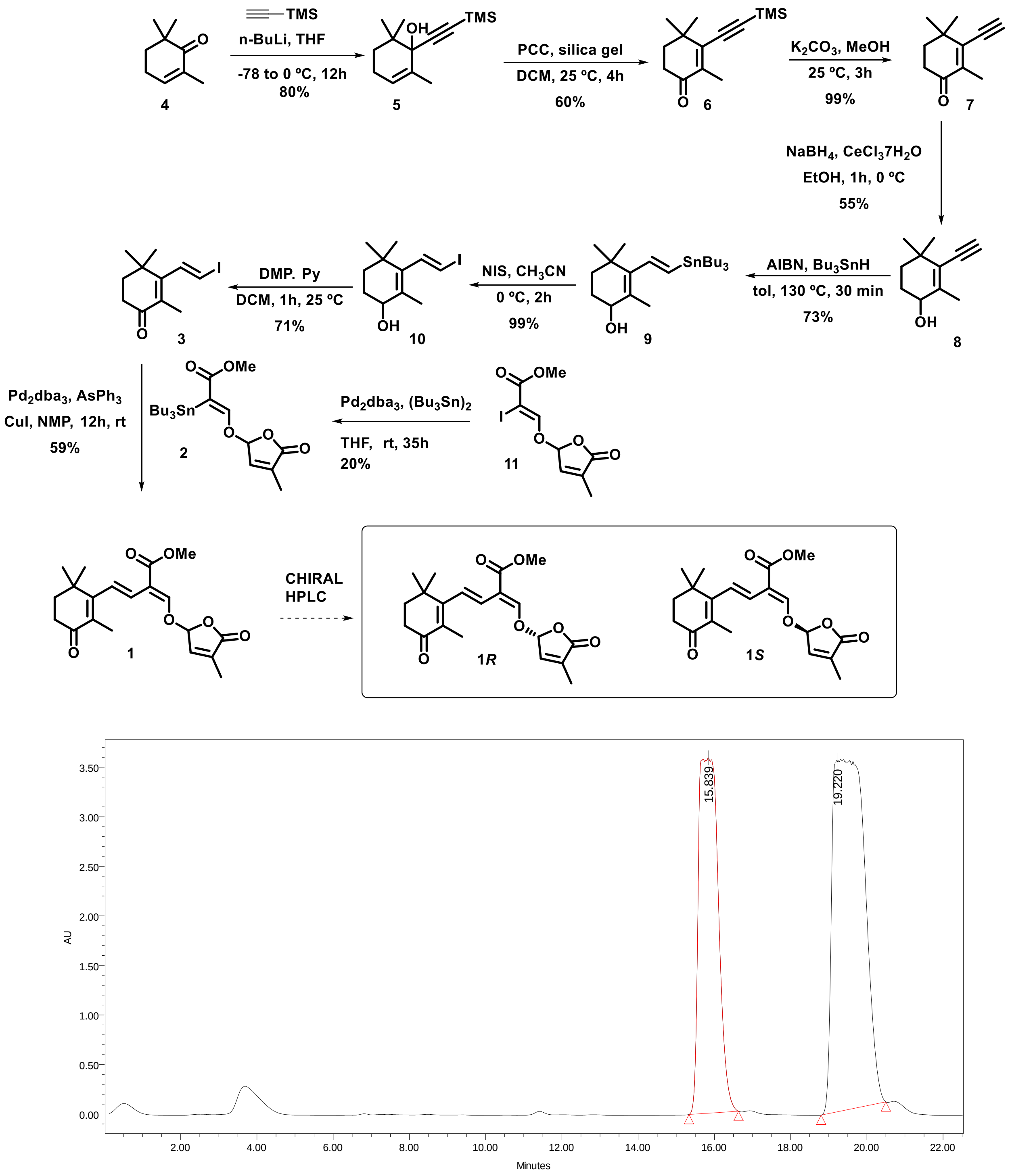

Fig. S1 Experimental procedures for the total synthesis of methyl 4-oxo-carlactonoate.

The synthesis of methyl 4-oxo-carlactonoate **1** (Scheme 1) started from 2,6,6-trimethylcyclohex-2-en-1-one **4**,[1] which was converted in 80% yield into the propargylic alcohol **5**[2] upon addition of the anion

generated by treatment of trimethylsilylacetylene with *n*-BuLi. Oxidative rearrangement in the presence of PCC and silica gel generated protected enynone **6**[2] in 60% yield. Alkyne deprotection was carried out under basic conditions to provide enynone **7**. Given the undesired regioselectivity upon attempted hydrostannylation,[3] reduction of enone **7**[2] using Luche´s protocol was required to obtain enynol **8**[3] in 55% yield. Radical hydrostannylation of **8** using *n*-Bu_3SnH in the presence of AIBN provided regio- and stereoselectively (*E*)-dienylstannane **9**[3] in 73% yield. Tin/iodine exchange was carried out using *N*-iodosuccinimide and afforded **10**[2] in quantitative yield. Oxidation of the secondary alcohol **10** upon treatment with Dess-Martin periodinane reagent in the presence of pyridine, afforded iododienylcyclohexone **3**[3] in 71% yield. In our experience, the Stille-Migita-Kosugi cross-coupling reaction[4-6] was best performed with alkenylstannane **2**,[7] generated by iodine-tin exchange from the corresponding alkenyl iodide **11**,[8] which reacted at ambient temperature with **3**[3] upon addition of catalytic amounts of Pd_2dba_3 and $AsPPh_3$ with CuI as additive,[7] and afforded *rac*-methyl 4-oxo-carlactanoate **1** in 59% yield. Both enantiomers were separated using chiral HPLC (Chiralpak IK SFC 5 cm; 1 x 25 cm; 66:17:17 *v/v/v* hexane/CH_2Cl_2/IPA; flow rate: 2 mL/min; 270 nm).

2,6,6-Trimethyl-1-((trimethylsilyl)ethynyl)cyclohex-2-en-1-ol (5). *n*-BuLi (6.42 mL, 13.17 mmol) was added to a cooled (0 ºC) solution of trimethylsilylacetylene (1.9 mL, 10.13 mmol) in THF (14 mL), and the mixture was stirred for 30 min. Then, it was cooled down to -78 ºC and a solution of 2,6,6-trimethylcyclohex-2-en-1-one **4** (1.4 g, 10.13 mmol) in THF (28 mL) was added. After stirring overnight at 0 ºC, water was added, and the resulting mixture was acidified with 10% HCl until pH 7. The residue was extracted with Et_2O (3x). The combined organic layers were washed with brine, dried and concentrated. The residue was purified by flash-column chromatography (silica gel, from 98:2 *v/v* *n*-hexane/Et_3N to 95:5 *v/v* *n*-hexane/EtOAc) to afford 1.92 g (80% yield) of a yellow oil, which was identified as compound **5**. The spectroscopic data matched those for the same product previously reported in the literature.[2] **^{1}H-NMR** (400.13 MHz, C_6D_6): δ 5.40 – 5.19 (m, 1H), 1.97 (td, *J* = 2.1, 1.4 Hz, 3H), 1.82 (ddt, *J* = 6.0, 3.8, 2.1 Hz, 2H), 1.54 (td, *J* = 6.4, 4.6 Hz, 2H), 1.20 (s, 3H), 1.13 (s, 3H), 0.15 (s, 9H) ppm.

2,4,4-Trimethyl-3-((trimethylsilylethynyl)cyclohex-2-en-1-one (6). To a magnetically stirred slurry of 1:1 PCC (3.75 g, 17.05 mmol) and silica gel (3.75 g, 62.43 mmol) in CH_2Cl_2 (34 mL), propargylic alcohol **5** (1.92 g, 8.12 mmol) in CH_2Cl_2 (17 mL) was added. The resulting mixture was stirred for 2h at room temperature. Once complete, the reaction mixture was filtered through a pad of silica gel and the solvent was concentrated. The residue was purified by flash-column chromatography (silica gel, 95:5 *v/v* *n*-hexane/EtOAc) to afford 1.07 g (56% yield) of a yellow oil, which was identified as compound **6**. The spectroscopic data matched those for the same product previously reported in the literature.[2] **^{1}H-NMR** (400.13 MHz, C_6D_6): δ 2.26 – 2.18 (m, 5H), 1.40 – 1.31 (m, 2H), 1.06 (s, 6H), 0.16 (s, 9H) ppm.

3-Ethynyl-2,4,4-trimethylcyclohex-2-en-1-one (7). To a solution of **6** (1.07 g, 4.57 mmol) in MeOH (29 mL), K_2CO_3 (1.26 g, 9.13 mmol) was added, and the mixture was stirred for 3h at room temperature. EtOAc was added, the layers were separated, and the organic layer was washed with a saturated aqueous solution of NH_4Cl and with brine. The organic layer was dried and concentrated. The yellow oil

residue (0.73 g, 99% yield), which was identified as compound **7**, was used in the next step without further purification. The spectroscopic data matched those for the same product previously reported in the literature.[2] **^{1}H-NMR** (400.13 MHz, C_6D_6): δ 3.14 – 3.12 (m, 1H), 2.23 – 2.15 (m, 2H), 2.14 (s, 3H), 1.35 – 1.30 (m, 2H), 0.98 (s, 6H) ppm.

3-Ethynyl-2,4,4-trimethylcyclohex-2-en-1-ol (8). To a solution of **7** (0.78 g, 4.81 mmol) in EtOH (50 mL), $CeCl_3 \cdot 7H_2O$ (2.22 g, 5.96 mmol) was added. The mixture was cooled down to 0 ºC and $NaBH_4$ (0.18 g, 4.81 mmol) was added. The reaction mixture was stirred for 1.5h at 0 ºC. A saturated aqueous solution of NH_4Cl was added and the mixture was extracted with Et_2O (3x). The combined organic layers were washed with brine, dried and concentrated. The residue was purified by flash-column chromatography (silica gel, 80:20 *v/v* *n*-hexane/EtOAc) to afford 0.43 g (55% yield) of a yellow oil, which was identified as compound **8**. The spectroscopic data matched those for the same product previously reported in the literature.[3] **^{1}H-NMR** (400.13 MHz, C_6D_6): δ 3.65 (s, 1H), 2.63 (s, 1H), 2.01 (s, 3H), 1.60 – 1.53 (m, 1H), 1.50 – 1.40 (m, 2H), 1.24 – 1.19 (m, 1H), 1.18 (s, 3H), 1.10 (s, 3H) ppm.

(*E*)-2,4,4-Trimethyl-3-(2-tributylstannyl)vinyl)cyclohex-en-1-ol (9). To a solution of **8** (0.43 g, 2.62 mmol) in toluene (37 mL), AIBN (43 mg, 0.26 mmol) and *n*-Bu_3SnH (2.8 mL, 10.47 mmol) were added, and the resulting mixture was stirred at 130 ºC for 45 min. After cooling down to room temperature, the solvent was concentrated. The residue was purified by flash-column chromatography (silica gel, 98:2 *v/v* *n*-hexane/Et_3N to 80:20 *v/v* *n*-hexane/EtOAc) to afford 0.87 g (73% yield) of a yellow oil, which was identified as compound **9**. The spectroscopic data matched those for the same product previously reported in the literature.[3] **^{1}H-NMR** (400.13 MHz, C_6D_6): δ 6.67 (d, *J* = 19.7 Hz, 1H), 6.14 (d, *J* = 19.7 Hz, 1H), 3.86 – 3.79 (m, 1H), 1.94 (s, 3H), 1.80 – 1.68 (m, 2H), 1.68 – 1.59 (m, 6H), 1.46 – 1.36 (m, 6H), 1.34 – 1.25 (m, 2H), 1.10 (s, 3H), 1.05 (s, 3H), 1.04 – 0.99 (m, 6H), 0.95 (t, *J* = 7.3 Hz, 9H) ppm.

(*E*)-3-(2-Iodovinyl)-2,4,4-trimethylcyclohex-2-en-1-ol (10). To a cooled (0 ºC) solution of **9** (0.87 g, 1.90 mmol) in CH_3CN (56 mL), NIS (0.56 g, 2.48 mmol) was added, and the resulting mixture was stirred at 0 ºC for 45 min. A mixture of saturated solutions of $NaHCO_3$ and $Na_2S_2O_3$ (2:1, *v/v*) was added and the mixture was extracted with Et_2O (3x). The combined organic layers were washed with brine, dried and concentrated. The residue was purified by flash-column chromatography (silica gel, 98:2 *v/v* *n*-hexane/Et_3N to 80:20 *v/v* *n*-hexane/EtOAc) to afford 0.56 g (99%) of a yellow oil, which was identified as compound **10**. The spectroscopic data matched those for the same product previously reported in the literature.[2] **^{1}H-NMR** (400.13 MHz, C_6D_6): δ 6.88 (dt *J* = 14.9, 1.2 Hz,1H), 5.82 (d, *J* = 14.8 Hz, 1H), 3.98 (s, 1H), 1.60 (d, *J* = 1.0 Hz, 3H), 1.58 – 1.49 (m, 1H), 1.49 – 1.35 (m, 2H), 1.12 (ddd, *J* = 13.1, 7.8, 3.1 Hz, 1H), 0.81 (s, 3H), 0.74 (s, 3H) ppm.

(*E*)-3-(2-Iodovinyl)-2,4,4-trimethylcyclohex-2-en-1-one (3). To a cooled (0 ºC) solution of **10** (50 mg, 0.17 mmol) in CH_2Cl_2 (1.9 mL), were sequentially added pyridine (0.069 mL, 0.86 mmol) and Dess-Martin Periodinane (145.2 mg, 0.34 mmol). The mixture was stirred for 1h at room temperature. Et_2O and a saturated aqueous solution of $NaHCO_3$ and $Na_2S_2O_3$ (1:1, *v/v*) were added. Then, the mixture was extracted with Et_2O (3x) and the combined organic layers were washed with brine, dried and

concentrated. The residue was purified by flash-column chromatography (silica gel, 98:2 *v/v* *n*-hexane/Et_3N to 90:10 *v/v* *n*-hexane/EtOAc) to afford 33.4 mg (71% yield) of a yellow oil, which was identified as compound **3**. The spectroscopic data matched those for the same product previously reported in the literature.[2] **^{1}H-NMR** (400.13 MHz, C_6D_6): δ 6.75 (dd, *J* = 15.0, 1.1 Hz, 1H), 5.82 (d, *J* = 15.0 Hz, 1H), 2.22 - 2.15 (m, 2H), 1.74 (d, *J* = 1.1 Hz, 3H), 1.28 – 1.23 (m, 2H), 0.63 (s, 6H) ppm.

Methyl (*Z*)-3-((4-Methyl-5-oxo-2,5-dihydrofuran-2-yl)oxy)-2-(tributylstannyl)acrylate (2).

To a solution of methyl (*Z*)-2-iodo-3-((4-methyl-5-oxo-2,5-dihydrofuran-2-yl)oxy)acrylate **11**[8] in degassed THF (4.9 mL), Pd_2dba_3 (43 mg, 0.048 mmol) and $(Bu_3Sn)_2$ (0.36 mL, 0.72 mol) were added at 0 ºC and the mixture was stirred at room temperature for 9h. Then, Pd_2dba_3 (43 mg, 0.048 mmol) was added at 0 ºC and the mixture was stirred at room temperature for 26h. The residue was filtered through Celite® and concentrated. The residue was purified by flash-column chromatography (silica gel, from 100:0 to 80:20 *v/v* *n*-hexane/EtOAc) to afford 0.046 g (20% yield) of a yellow oil, which was identified as compound **2**. The spectroscopic data matched those for the same product previously reported in the literature.[7] **^{1}H-NMR** (400.13 MHz, $CDCl_3$): δ 8.09 (s, 1H), 5.95 – 5.80 (m, 1H), 5.17 (t, *J* = 1.5 Hz, 1H), 3.57 (s, 3H), 1.80 – 1.67 (m, 6H), 1.52 – 1.45 (m, 9H), 1.31 – 1.22 (m, 6H), 1.04 (t, *J* = 7.3 Hz, 9H) ppm.

Methyl (2*E*,3*E*)-2-(((4-Methyl-5-oxo-2,5-dihydrofuran-2-yl)oxy)methylene-4-(2,6,6-trimethyl-3-oxocyclohex-1-en-1-yl)but-3-enoate (1). To a solution of **3** (30 mg, 0.10 mmol) in NMP (1 mL), stannane **2** (58 mg, 0.12 mmol), Pd_2dba_3 (10 mg, 0.01 mmol), $AsPh_3$ (13 mg, 0.041 mmol) and CuI (20 mg, 0.10 mmol) were sequentially added. The mixture was stirred for 12h at room temperature. Water was added and the resulting mixture was extracted with Et_2O (3x). The combined organic layers were washed with brine, dried and concentrated. The residue was purified by flash-column chromatography (silica gel, 98:2 *v/v* *n*-hexane/Et_3N to 50:50 *v/v* *n*-hexane/EtOAc) to afford 22.1 mg (59% yield) of a yellow oil, which was identified as compound **1**. **^{1}H-NMR** (400.13 MHz, C_6D_6): δ 7.49 (s, 1H), 7.39 (d, *J* = 16.7 Hz, 1H), 6.59 (d, *J* = 16.7 Hz, 1H), 5.72 (s, 1H), 5.10 (s, 1H), 3.40 (s, 3H), 2.32 (dd, *J* = 7.5, 6.2 Hz, 2H), 2.14 (s, 3H), 1.47 – 1.40 (m, 2H), 1.33 (s, 3H), 0.97 (s, 3H), 0.93 (s, 3H) ppm. **^{13}C NMR** (100.62 MHz, C_6D_6): δ 197.5 (s), 169.7 (s), 166.1 (s), 160.4 (s), 153.8 (d); 140.5 (d), 135.3 (s), 130.7 (d), 130.1 (s), 124.8 (d), 112.2 (s), 100.4 (d), 51.3 (q), 37.5 (t), 35.6 (s), 34.6 (t), 27.4 (q, 2x), 14.2 (q), 10.2 (q) ppm. **HRMS (ESI$^+$)**: Calcd. for $C_{20}H_{25}O_6$ [$(M+H)^+$], 361.1645, found 361.1647. **IR** (NaCl): υ 2955 (m), 2278 (m), 1660 (m), 1786 (s, C=O), 1716 (s, C=O), 1660 (s, C=O), 1353 (w), 1194 (w), 1013 (w), 952 (w) cm^{-1}. Peak 1 HPLC: (*R*)-Methyl-4-oxocarlactonoate $[\alpha]_D^{22}$ +147.2 (*c* 0.28, CH_2Cl_2). Peak 2 HPLC: (*S*)-Methyl-4-oxocarlactonoate $[\alpha]_D^{23}$ -133.1 (*c* 0.34, CH_2Cl_2).

Column: Chiralpak IK 1x25 cm, hexane/CH_2Cl_2/IPA 66:17:17 v/v/v; flow rate: 2 mL/min, detection at 270 nm.

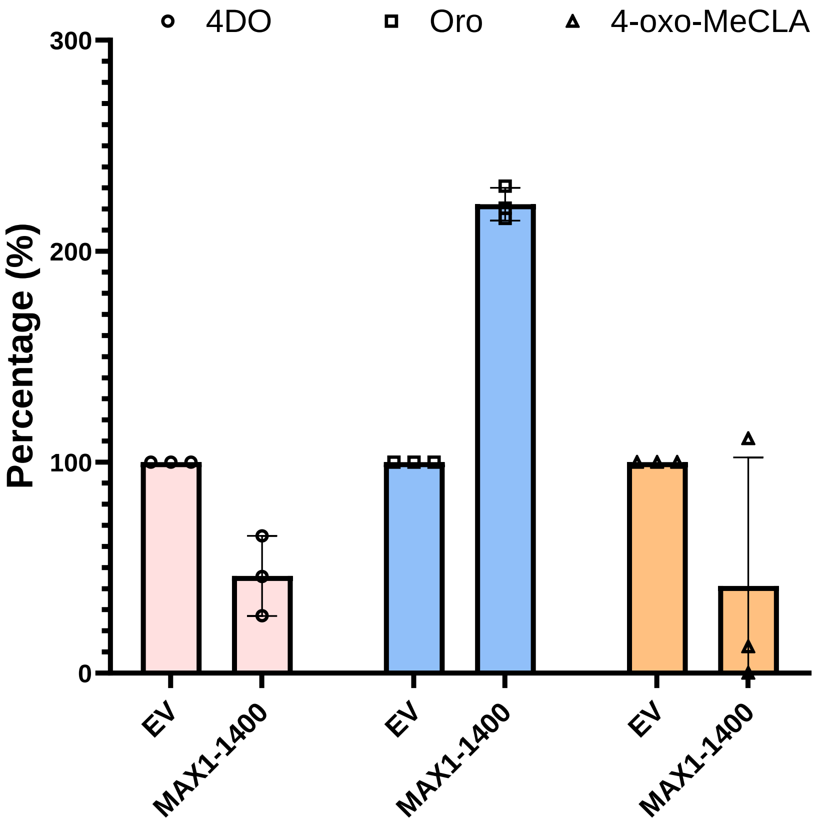

Fig. S2 MAX1-1400 potentially metabolizes both canonical and non-canonical SLs.

The levels of metabolites in root exudates feeding experiments with recombinant MAX1-1400 in yeast microsomes. The data are presented as means ± SD of 3 biological replicates.

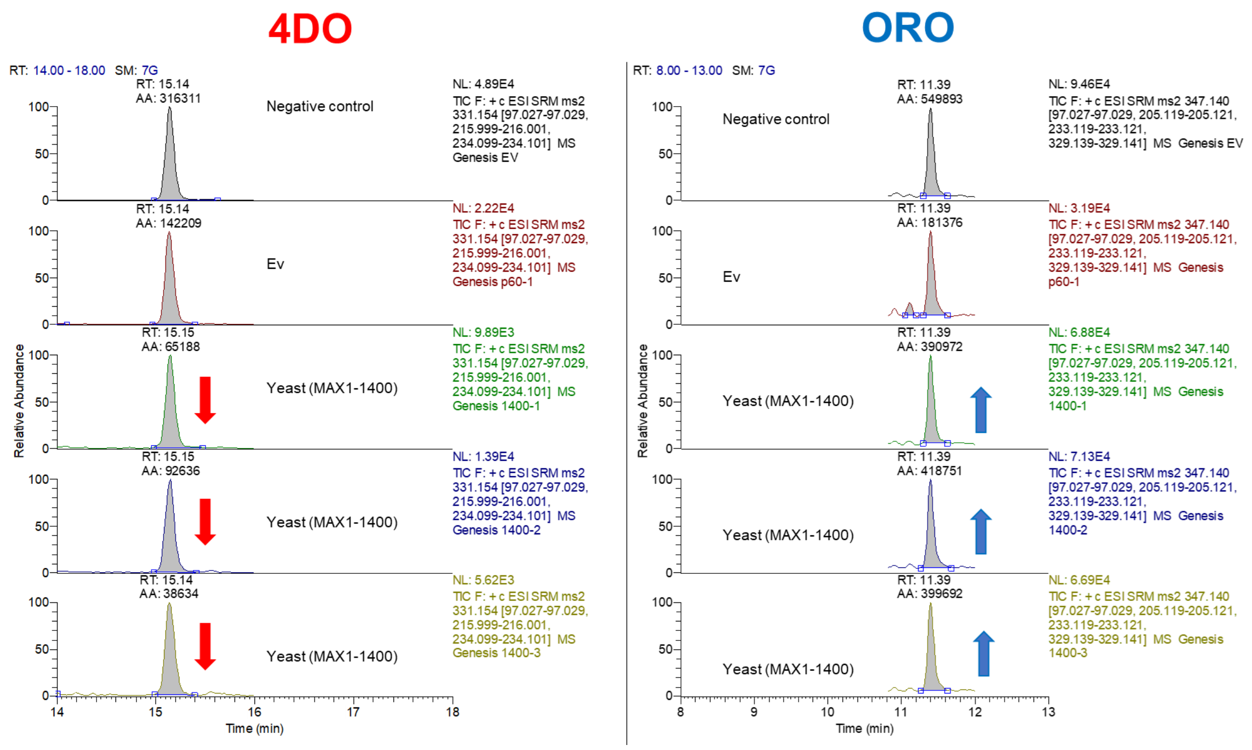

Fig. S3 Detection of 4DO and Oro in root exudates feeding experiments with recombinant MAX1-1400 in yeast microsomes.

Multiple reaction monitoring (MRM) was used to identify the levels of SLs. Chromatograms of 4DO and Oro detected in empty vector (EV) and yeast microsomes expressed MAX1-1400. The red arrows indicate decreased peak area while blue arrows indicate increased peak area compared to EV.

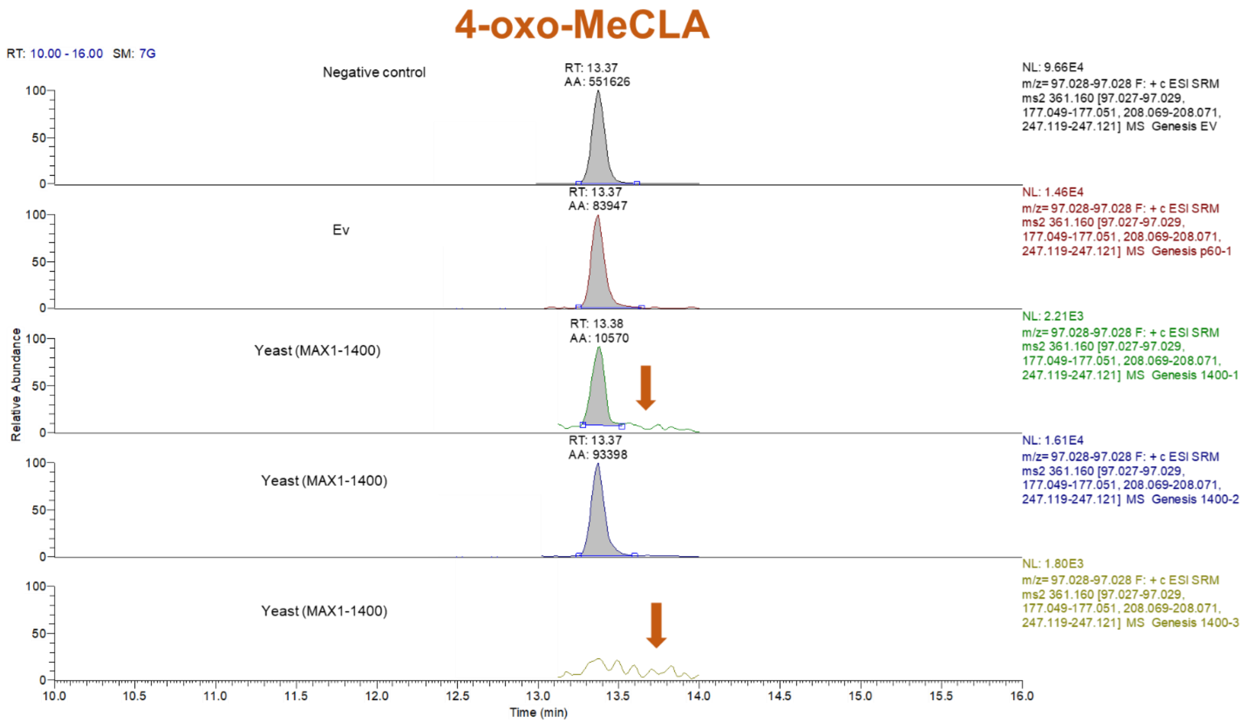

Fig. S4 Detection of 4-oxo-MeCLA in root exudates feeding experiments with recombinant MAX1-1400 in yeast microsomes.

Multiple reaction monitoring (MRM) was used to identify the levels of SLs. Chromatograms of 4-oxo-MeCLA detected in empty vector (EV) and yeast microsomes expressed MAX1-1400. The brown arrows indicate decreased peak area in comparison to EV.

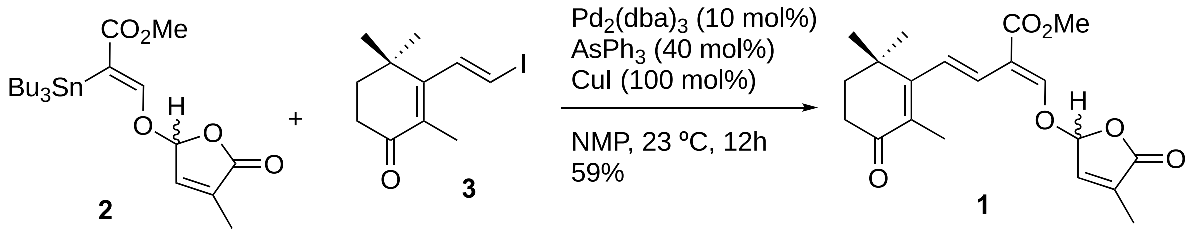

Fig. S5 Synthesis of methyl 4-oxo-carlactonoate (4-oxo-MeCLA).

Methyl 4-oxo-carlactonoate (**1**) was prepared from previously described alkenylstannane (**2**) and alkenyl iodide (**3**), by the Stille-Migita-Kosugi cross-coupling reaction, which took place at ambient temperature upon addition of catalytic amounts of Pd_2dba_3 and $AsPPh_3$ with CuI as additive, in 59% yield. Both enantiomers were separated using chiral HPLC (Chiralpak IK SFC 5 cm; 1 x 25 cm; 66:17:17 v/v/v hexane/CH_2Cl_2/IPA; flow rate: 2 mL/min; 270 nm). Detailed experimental procedures are listed below.

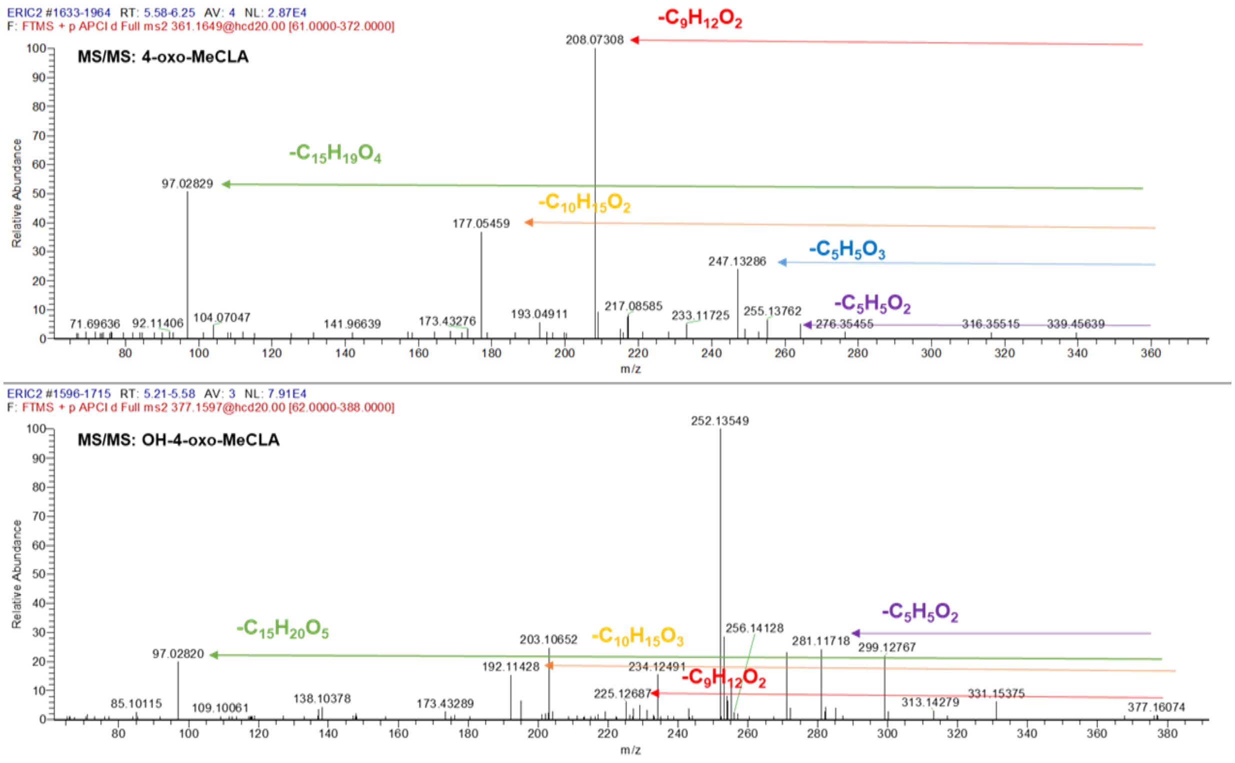

Fig. S6 Identification of OH-4-oxo-MeCLA.

Detection of OH-4-oxo-MeCLA in 4-oxo-MeCLA feeding experiments with recombinant MAX1-1400. The product ion spectra derived from the precursor ion of m/z 361.1649 [M+H]+ and m/z 377.16074 [M+H]+ in positive mode by Collision Energy (CE) 20 V were shown. The loss of mass difference was calculated by Xcalibur software version 4.1. Mass tolerance: ± 5 ppm

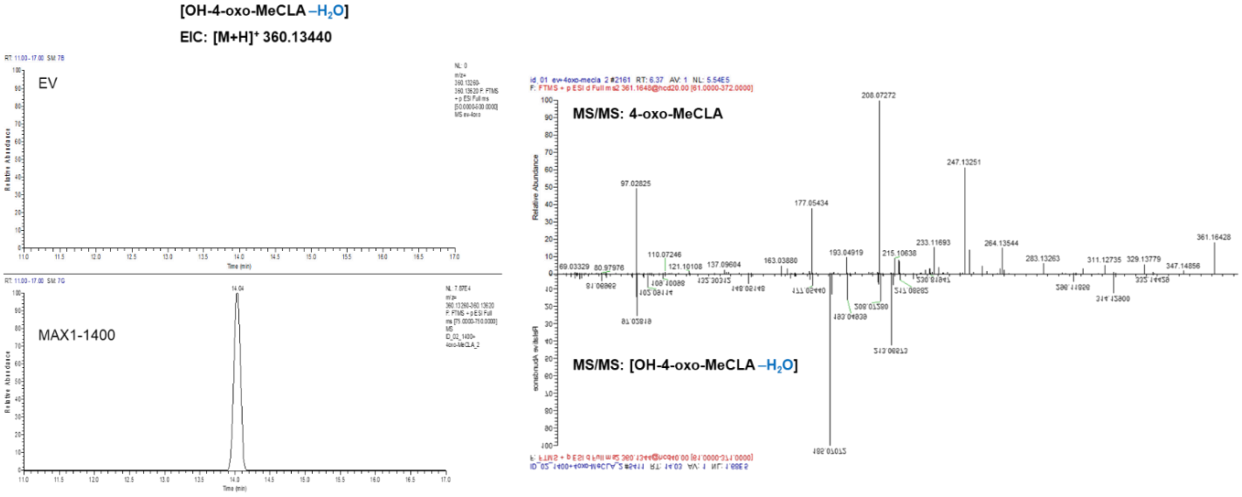

Fig. S7 Detection of OH-4-oxo-MeCLA in 4-oxo-MeCLA feeding experiments with recombinant MAX1-1400.

Identification of OH-4-oxo-MeCLA, based on accurate mass (Left) and MS/MS pattern (Right), in comparison to empty vector and authentic 4-oxo-MeCLA standard. Mass tolerance: ± 5 ppm

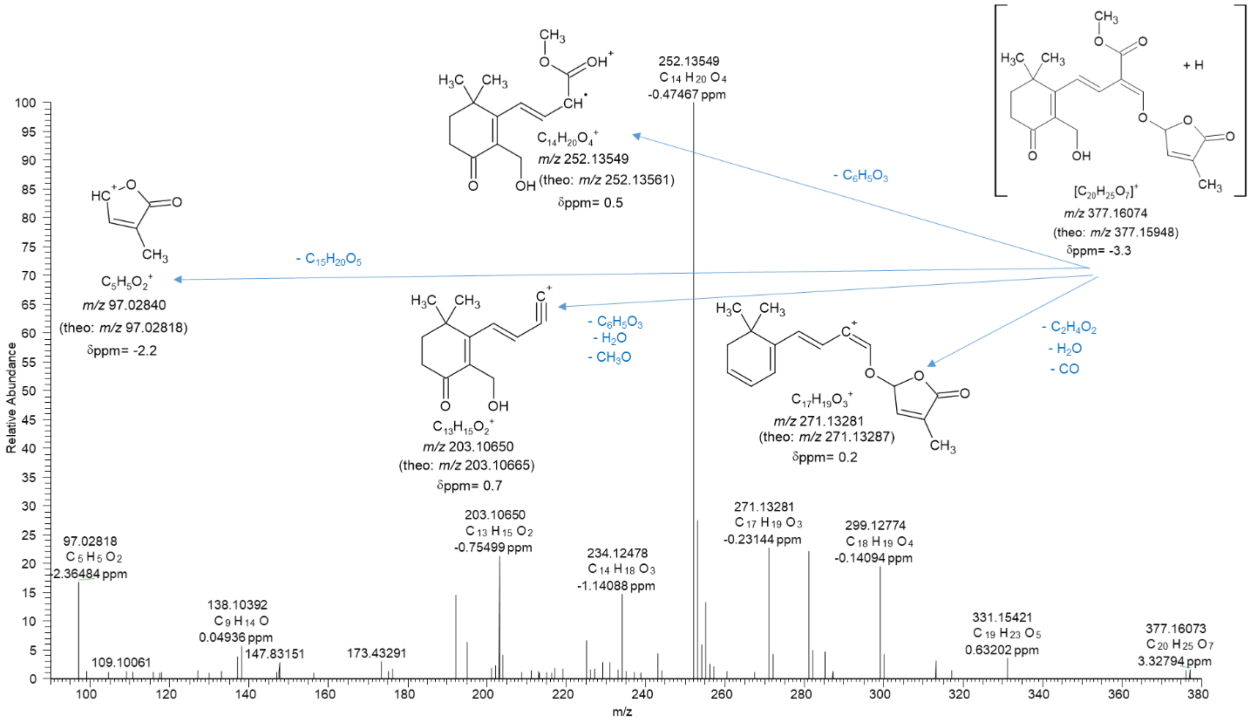

Fig. S8 Proposed structure of tentative 18-OH-4-oxo-MeCLA based on mass fragmentation.

Proposed structure of 18-OH-4-oxo-MeCLA ($[M+H]^+$ 377.16074 in positive mode) was calculated from MS/MS fragmentation, identified from recombinant MAX1-1400 in yeast microsomes fed with 4-oxo-MeCLA.

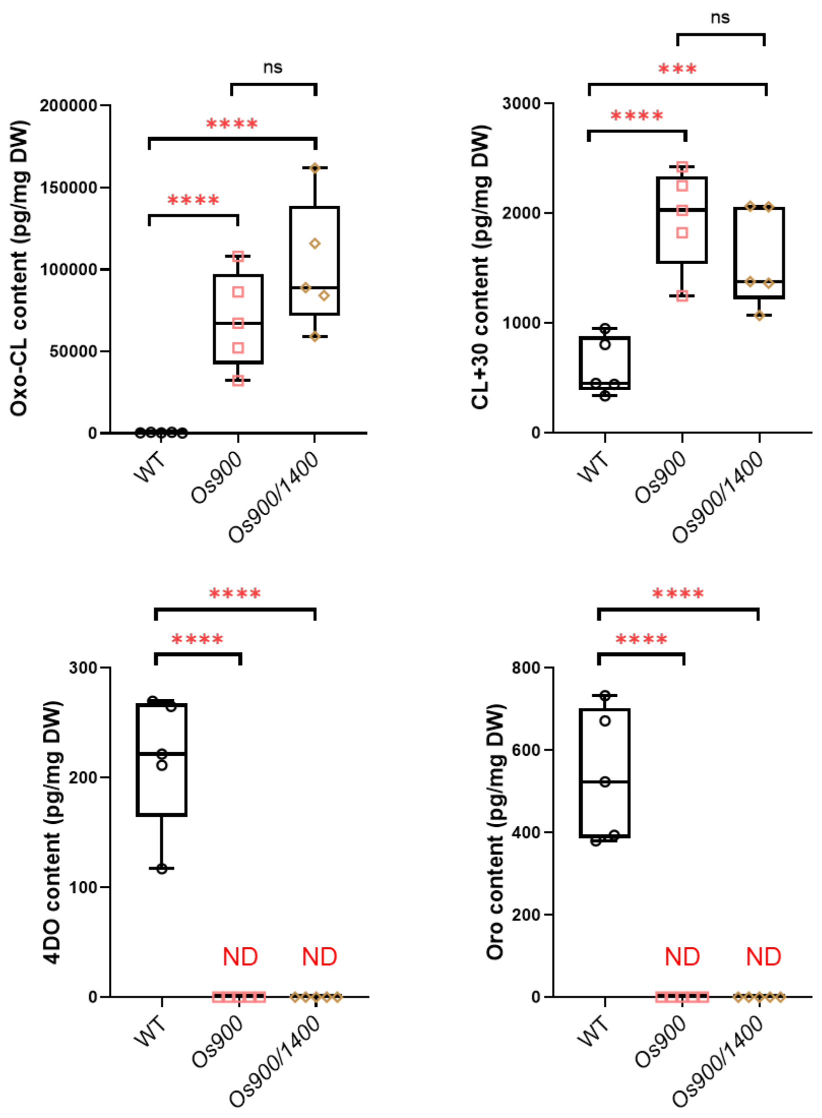

Fig. S9 SL quantification of Os900-KO line and Os900/1400-KO mutants.

Analysis of SLs in root exudates of WT, *Os900*, and *Os900/1400* lines grown under constant low-Pi conditions. The data are presented as means ± SD of 5 biological replicates. Significant values determined by one-way ANOVA are shown with different letters ($P < 0.05$) when compared to WT, and asterisks indicate statistically significant differences as compared to control by the two tailed unpaired Student t test (*$P < 0.05$; **$P < 0.01$; ***$P < 0.001$; ****$P < 0.0001$). Abbreviations: CL, carlactone, 4DO, 4-deoxyorobanchol, Oro, orobanchol, WT, wild-type; ND, not detected; ns, non-significant.

SI REFERENCES

1. W. Oppolzer, T. Sarkar and K. K. Mahalanabis, A Simple Alkylative 1,2-Carbonyl Transposition of Cyclohexenones, *Helv. Chim. Acta*, 1976, **59**, 2012-2020.
2. M. Domínguez, R. Alvarez, S. Martras, J. Farrés, X. Parés and A. R. de Lera Synthesis of Ring-oxidized Retinoids as Substrates of Mouse Class I Alcohol Dehydrogenase (ADH1), *Org. Biomol. Chem.*, 2004, **2**, 3368-3373.
3. M. Domínguez, S. Álvarez, R. Álvarez and Å. R. de Lera, Stereocontrolled synthesis of (S)-9-cis-4-oxo-13,14-dihydroretinoic acid, *Tetrahedron*, 2012, **68**, 1756-1761.
4. J. K. Stille, The Palladium-Catalyzed Cross-Coupling Reactions of Organotin Reagents with Organic Electrophiles, *Angew. Chem., Int. Ed. Engl.*, 1986, **25**, 508-524.
5. V. Farina and G. P. Roth, *Recent Advances in the Stille Reaction*, JAI Press, Greenwich, CT, 1996.
6. M. M. Heravi, E. Hashemi and F. Azimian, Recent developments of the Stille reaction as a revolutionized method in total synthesis, *Tetrahedron*, 2014, **70**, 7-21.
7. S. Woo and C. S. P. McErlean, Total Synthesis and Stereochemical Confirmation of Heliolactone, *Org. Lett.*, 2019, **21**, 4215-4218.
8. M. C. Dieckmann, P.-Y. Dakas and A. De Mesmaeker, Synthetic Access to Noncanonical Strigolactones: Syntheses of Carlactonic Acid and Methyl Carlactonoate, *J. Org. Chem*, 2018, **83**, 125-135.
